# Science and innovation in Sub-Saharan Africa: an introduction

**DOI:** 10.1098/rsfs.2024.0020

**Published:** 2024-08-09

**Authors:** C. Richard A. Catlow, John Pickett

**Affiliations:** ^1^ School of Chemistry, University of Cardiff, Park Place, Cardiff CF10 3AT, UK; ^2^ Department of Chemistry, University College London, 20 Gordon Street, London WC1H 0AJ, UK

**Keywords:** science, innovation, Sub-Saharan Africa

The development of a strong capacity for scientific research and innovation in Africa is a key priority for the African continent and indeed for the world. Its importance was recognized over 30 years ago by the Royal Society, and in 2012, following a successful previous programme largely based on research student training and funded by the Leverhulme Trust, the Society was granted funding by the UK Department for International Development to develop a capacity-strengthening programme to be based in Sub-Saharan Africa. The programme—the Africa Capacity Building Initiative (ACBI)—was based on consortia involving scientists and engineers from UK universities and counterparts in African universities and research institutes who worked to build research capacity in key areas, including sustainable energy, water and sanitation and soil-related research. It aimed to build sustainable partnerships, to strengthen the research training capacity in Sub-Saharan African universities and to train a cohort of talented early career researchers by a PhD scholarship programme. Each project received funding towards PhD studentships, research expenses, travel and subsistence costs, training and limited funds for equipment. The initial phase of funding allowed consortia to meet and develop their plans for the main tranche of funding, starting in 2014. Many high-quality applications were received of which 10 were selected for funding by a panel chaired by one of us (J.P.). Panel members, together with Royal Society staff monitored progress, which was helped by the plenary meetings of the whole programme (see figure 1). Additional support and advice were provided by the Centre for Capacity Research at the Liverpool School of Tropical Medicine.

**Figure 1.**
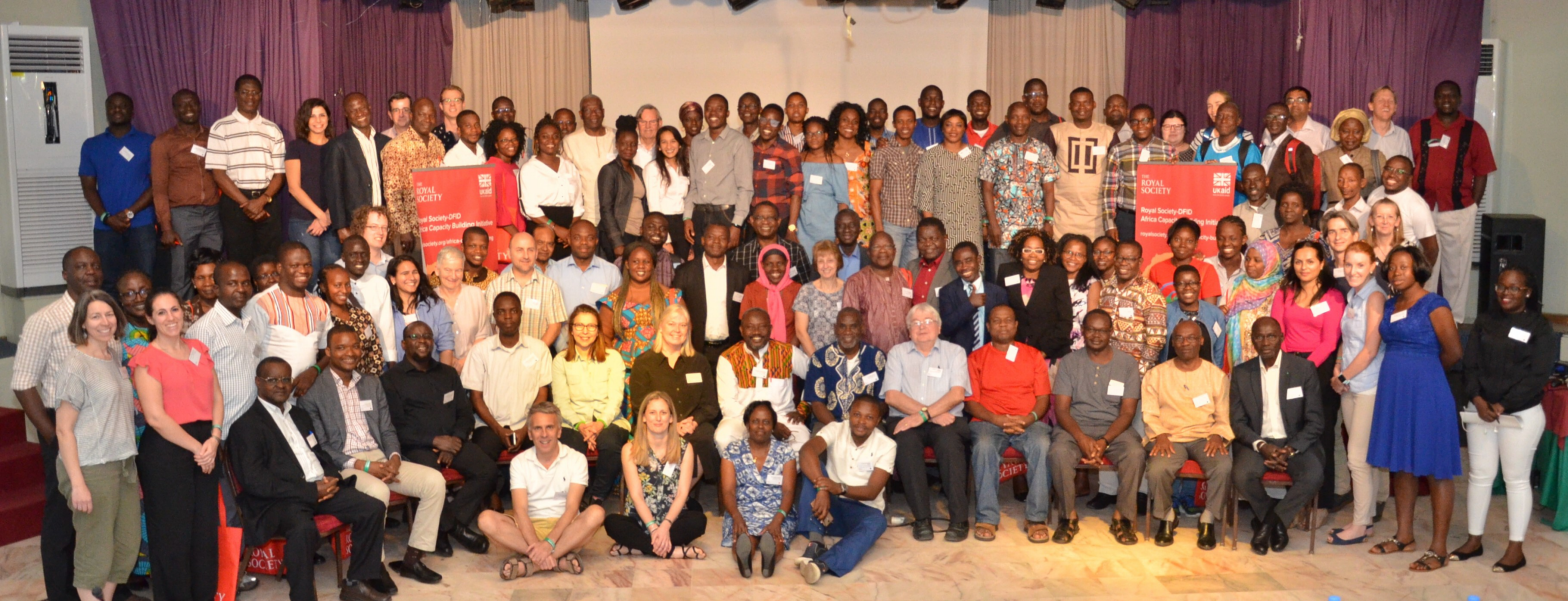
ACBI Consortia Meeting held in M’Bour, Senegal, December 2018.

Seven of the articles in the issue are based on ACBI consortia. They illustrate the range of science and engineering encompassed by the programme and describe the successes in institutional capacity strengthening and student development and training. They also show how the aim of establishing sustainable partnerships was in many cases achieved and that high-quality scientific outputs were produced.

The first four articles focus on the theme of sustainable energy, with the first by Ungerer *et al.* [1] summarizing the achievements of the ‘Chem4Energy’ programme, which brought together teams in Cardiff University, UK, with those in four African universities: Kwame Nkrumah University of Science and Technology (KNUST) in Ghana, the University of Namibia, the University of Botswana and Botswana International University of Science and Technology (BIUST); the wide-ranging programme exploited the synergy between experiment and computational modelling to explore new materials for sustainable energy technologies. The consortium remains active and holds an annual conference.

The article of Langmi *et al.* [2] reviews the achievements of the consortium comprising the University of Nottingham, UK (and the University of Nottingham, Ningbo, China), the University of Yaounde, Cameroon, the University of Pretoria, South Africa, and Maseno University, Kenya; their programme focused on the development of new porous materials for sustainable energy applications relating to hydrogen storage and valorization of biomass to renewable fuels. They also succeeded in preparing porous composite materials which could be used in electrochemical sensing.

Ndifon *et al*. [3] discuss the work of the ‘CaGSUMI’ consortium involving Manchester University, UK, working with KNUST (Ghana), the University of Zululand, South Africa, and the University of Yaounde, which again focused on materials chemistry for renewable energy technologies, especially solar cells, with a strong emphasis on nano-chemistry and thin-film technologies. It also succeeded in installing crucial equipment necessary for competitive work in this field in several African universities.

In the review of Winchester *et al*. [4] the emphasis is on new developments in engineering science undertaken by a consortium comprising Imperial College London with the Universities of Lagos, Nigeria, and the Universities of Pretoria and Mauritius, who worked on direct-steam generation of concentrated solar power (CSP) plants. Among other achievements, their work predicted an ~80% increase in the net present value of a case-study CSP plant.

The focus then moves to soil science in the article of Manzeke-Kangara *et al.* [5] describing the work of a consortium bringing together expertise in the Universities of Nottingham, the British Geological Survey, Lilongue and Mzuzu Universities, Malawi, the Ministry of Health, Malawi, Zambia Agricultural Research Institute, the University of Zambia and the University of Zimbabwe to examine the linkages between soil geochemistry, agriculture and public health to enhance crop productivity, nutrition and food safety. Their work will contribute towards the development of a national micronutrient survey and has led to follow-up funding.

Logah *et al*. [6] continue the theme of soil science by discussing the work of a wide-ranging consortium including the Federal University of Agriculture, Nigeria, the International Institute of Tropical Agriculture, Nigeria, KNUST and the CSIR-Crops Research Institute Ghana, the Institut de l’Environnement et de Recherches Agricoles, Burkina Faso, the Hungarian University of Agriculture and Life Sciences, Wageningen University, The Netherlands, and Imperial College London, UK. The consortium examined the biogeochemistry of anthropogenically formed forest islands compared to adjacent natural savanna and croplands. They showed that the formation of forest islands is anthropogenically driven, and the consortium provided training in research relevant to climate change adaptation and food systems transformation.

Bates *et al*. [7] then explore river science relating to the Congo Basin; their consortium comprised the University of Kinshasha, Democratic Republic of Congo, Ardhi University and the University of Dar Es Salaam, Tanzania, Rhodes University, South Africa, with the Universities of Leeds and Bristol, UK. Their aim was to improve understanding of the hydraulics and geomorphology of this globally important river, which is essential for navigation, irrigation, drinking water and hydroelectric power generation for 10 countries. Their achievements included helping to develop a new hydrology research centre at the University of Kinshasa and extending international involvement in Congo Basin research. This unique investigation, often conducted under severely difficult safety concerns, may offer wider knowledge of how such river systems develop after intense infrastructure support for management ceases and how to rebuild hydrology management more sustainably and an international community for river basin research.

The final article of Ngoepe *et al*. [8] relates to an earlier initiative established in the 1990s shortly after the regime change in South Africa and whose aim was to develop research capacity in the historically disadvantaged universities in South Africa. The article describes the development of a successful, internationally competitive research centre in the field of computational materials modelling at the University of Limpopo (formerly the University of the North). It shows the success of the centre in terms of new science relevant to South African needs and the development of expertise in this key field.

Overall, the articles in this issue show the value impact and success of capacity-strengthening programmes particularly where science underpins the development opportunities. The projects described not only led to trained cohorts of early career scientists (including 38 PhD graduates in the ACBI programme, from 26 African research institutions across 18 African countries) together with laboratory and institutional research capacity strengthening; but they also produced high-quality internationally competitive science as was confirmed by a review conducted by the Royal Society of the 357 research outputs submitted by the ACBI consortia.

In conclusion, we would like to thank Tim Holt and colleagues in the Royal Society publishing division for their assistance in producing this issue, and to Natasha Bevan and her colleagues in the Royal Society International Grants team. We are also grateful to all those who contributed to this issue and the ACBI and other Royal Society capacity-strengthening programmes.

## Data Availability

This article has no additional data.

## References

[1] Ungerer MJ *et al*. 2024 Chem4Energy: a consortium of the Royal Society Africa Capacity-Building Initiative dedicated to the memory of Professor Richard Tia. Interface Focus **14**, 20240001. (10.1098/rsfs.2024.0001) 39129852 PMC11310705

[2] Langmi HW, Musyoka NM, Kemmegne-Mbouguen JC, Kowenje C, Kengara F, Mokaya R. 2024 Capacity building in porous materials research for sustainable energy applications. Interface Focus **14**, 20230067. (10.1098/rsfs.2023.0067) 39129854 PMC11310698

[3] Ndifon PT, Awudza J, Revaprasadu N, O’Brien P, Lewis D. 2024 Portrait of a UK–Africa capacity building initiative consortium 2015–2022: the Cameroon, Ghana, South Africa and United Kingdom Materials Initiative (CaGSUMI) for developing materials for solar cells. Interface Focus **14**, 20230057. (10.1098/rsfs.2023.0057) 39129858 PMC11310707

[4] Winchester B, Maghrabi AM, Markides CN. 2024 RS–DFID Africa capacity-building initiative programme grant: harnessing unsteady phase-change heat exchange in high-performance concentrated solar power systems. Interface Focus **14**, 20230059. (10.1098/rsfs.2023.0059) 39129853 PMC11310711

[5] Manzeke-Kangara MG *et al*. 2024 Doctoral training to support sustainable soil geochemistry research in Africa. Interface Focus **14**, 20230058. (10.1098/rsfs.2023.0058) 39129856 PMC11310714

[6] Logah V, Azeez JO, Compaore H, Mesele SA, Ocansey CM, Bougma AB, Tetteh EN, Veenendaal E, Lloyd J. 2024 Exploring the West African forest island phenomenon: scientific insights gained, successes achieved and capacities strengthened. Interface Focus **14**, 20230078. (10.1098/rsfs.2023.0078) 39165392 PMC11331262

[7] Bates PD, Tshimanga RM, Trigg MA, Carr A, Mushi CA, Kabuya PM, Bola G, Neal J, Nbomba P. 2024 Creating sustainable capacity for river science in the Congo basin through the CRuHM project. Interface Focus **14**, 20230079. (10.1098/rsfs.2023.0079) 39129855 PMC11310709

[8] Ngoepe PE, Chadwick AV, Sithole HM, Mokhele KDK, Catlow CRA. 2024 Materials modelling in the University of Limpopo. Interface Focus **14**, 20240005. (10.1098/rsfs.2024.0005) 39129857 PMC11310708

